# A confidence-gated source selection strategy for cross-session transfer in brain–computer interfaces

**DOI:** 10.3389/fnhum.2026.1895016

**Published:** 2026-08-28

**Authors:** Yiming Shen, David Degras

**Affiliations:** Department of Mathematics, University of Massachusetts Boston, Boston, MA, United States

**Keywords:** brain-computer interface, confidence interval, cross-session transfer, longitudinal EEG, motor imagery EEG, multiple source domain adaptation, uncertainty-aware source selection

## Abstract

Cross-session variability remains a major obstacle to the reliable operation of motor imagery (MI)-based brain-computer interfaces (BCI), particularly when systems are reused across multiple days. When multiple prior sessions from the same subject are available, two key questions arise before domain transfer: which source sessions to select and how to effectively utilize them. We address these questions by developing confidence-gated, selective-transfer pipelines: a Minimum-Distance Multi-Source Pipeline (MMP) that only uses source sessions close to the target, and a Bridge Domain Pipeline (BDP) that exploits both near and far sources to improve robustness. We evaluated these novel pipelines against uniform-pooling (MAP) and distance-weighted-pooling (DWP) baseline methods on two public motor imagery EEG datasets under matched experimental configurations of feature extraction, classification, and domain adaptation algorithms. The benchmark results support an endpoint-specific interpretation rather than a single accuracy ranking. Specifically, MAP achieved the highest maximum-configuration accuracy, DWP demonstrated the highest average accuracy across configurations, and on the primary dataset MAP, DWP, and BDP exhibited no statistically significant differences as the top-performing pipelines for data-driven configuration selection. In contrast, MMP_mta_ performed similarly under fixed configurations but proved less effective during data-driven configuration selection. Overall, the best-performing proposed pipeline (BDP) ranks among the highest-performing approaches with respect to accuracy while requiring substantially reduced execution time and fewer source sessions than full pooling–demonstrating that BDP transforms the CI-gated retention idea into a more reliable and computationally efficient framework for automated configuration selection. These results indicate that in cross-session MI decoding, the primary challenge in selective transfer extends beyond session selection alone to encompass how retained sessions are integrated downstream, offering significant implications for longitudinal rehabilitation and assistive BCI use.

## 1 Introduction

Motor imagery-based brain-computer interfaces (MI-BCIs) decode imagined movements from non-invasive electroencephalography (EEG) signals to enable neural communication and device control without overt muscular activity. A persistent barrier to routine clinical and assistive deployment is that decoding models calibrated on a single recording session often suffer severe performance degradation when applied to subsequent sessions from the same subject. Changes in electrode impedance, physical montage displacement, fatigue, attention, and other session-dependent physiological or technical factors induce non-stationarity in the EEG signal distributions, substantially compromising cross-session decoding accuracy (Krauledat et al., 2008; Vidaurre et al., 2011; Samek et al., 2012; Raza et al., 2016). Consequently, this inter-session instability remains a major unresolved challenge to reliable MI-BCI operation beyond controlled laboratory environments.

Most methods address cross-session variability through domain adaptation (DA), which aligns source and target distributions before classification. Comprehensive reviews of EEG transfer learning and MI-BCI methods summarize this growing body of literature (Jayaram et al., 2016; Wan et al., 2021; Wu et al., 2022). Representative examples include Subspace Alignment, Transfer Component Analysis, Correlation Alignment (CORAL), and Riemannian transfer frameworks (Fernando et al., 2013; Pan et al., 2011; Sun et al., 2016; Zanini et al., 2018; Yger et al., 2017). Broader domain-adaptation paradigms encompass transferable-feature learning, adversarial adaptation, optimal transport, and multiple-source adaptation (Long et al., 2015; Ganin et al., 2016; Courty et al., 2017; Hoffman et al., 2018).

Other studies address non-stationarity through stationary spatial filtering, covariate-shift correction, or advanced signal processing techniques designed to minimize recalibration requirements (Samek et al., 2012; Raza et al., 2018; Lotte, 2015). Public datasets and the Mother of All BCI Benchmarks (MOABB) benchmarking framework now facilitate standardized, controlled cross-session comparisons (Jayaram and Barachant, 2018; Ma et al., 2022; Stieger et al., 2021; Yang et al., 2025). Broader benchmark references include the BCI Competition IV overview (Tangermann et al., 2012) and a large-scale MOABB reproducibility study (Chevallier et al., 2024). However, these studies predominantly focus on how to adapt a model; the prerequisite question of how to optimally structure and select among multiple available source sessions has received less direct attention.

For within-subject transfer, available non-target sessions can be uniformly pooled, weighted by domain distance, or pruned to a target-dependent subset. However, session selection introduces a secondary challenge: how should the retained sessions be leveraged to choose an optimal model configuration? Earlier multi-source BCI studies demonstrated that prior recordings can provide complementary rather than interchangeable information (Fazli et al., 2015; Liang et al., 2022). The most directly related study to our work evaluates relevant session selection for inter-session MI transfer (Sung et al., 2024). However, that study does not decouple CI-gated retention from configuration selection across a common feature–classifier–adaptation hyperparameter grid. Furthermore, research on negative transfer highlights that inaccurate relevance estimates or excessive pruning can eliminate beneficial training information (Zhang et al., 2023). In contrast, cross-subject source selection addresses a distinct problem wherein between-subject heterogeneity rather than within-subject, inter-session variance—serves as the primary driver of transfer degradation (Lee et al., 2024).

Source selection and configuration choice can affect the same accuracy result. An apparent gain may come from a favorable feature, classifier, or adaptation method rather than from the organization of prior sessions. A best-configuration summary can also exaggerate a difference because it assumes ideal tuning. We therefore evaluate every pipeline on the same feature–classifier–adaptation grid and report best-configuration, configuration-averaged, selected-configuration, and runtime summaries. MMP and BDP share one CI-gated near-set construction but use different proxy tasks to select a configuration. The comparison separates three stages: construction of the source set, selection of a model configuration, and fitting of the final model.

The study makes three contributions. First, it defines a confidence-gated source-selection rule for within-subject cross-session MI-BCI transfer. The rule uses bootstrap uncertainty in source-to-target distance to construct a target-dependent near set. Second, it defines two pipelines, MMP and BDP, that use this set differently when selecting a configuration. Third, it compares these pipelines with full-pooling and distance-weighted references on two public MI-EEG datasets. The benchmark reports best-configuration, configuration-averaged, and pipeline-selected accuracy separately, together with source use and runtime. Our expectation is that source selection strategies can achieve accuracy comparable to full data pooling while using less data, thus leading to storage and computational gains.

Section 2 defines the source-selection rule, the four pipeline families, and the shared feature, classifier, and adaptation grid. Section 3 reports the benchmark results. Section 4 discusses their interpretation and limitations.

## 2 Materials and methods

### 2.1 Problem setting and notation

We use within-subject leave-one-session-out evaluation. For one subject and one fold, let $S=\left\{S_{1},\ldots ,S_{K}\right\}$ be the set of *K* labeled non-target sessions, where *S*_*k*_ denotes source session *k*. Let *T* be the held-out target session. Let C denote the common set of 24 candidate model configurations, where each configuration specifies one feature representation, classifier, and domain-adaptation option. Target labels are used only to compute final test accuracy. Unlabeled target data can be used for domain adaptation and for source-to-target distance calculations. The comparison therefore concerns source selection and model selection within the same subject.

### 2.2 Source-selection and configuration-selection pipelines

**Figure 1:**
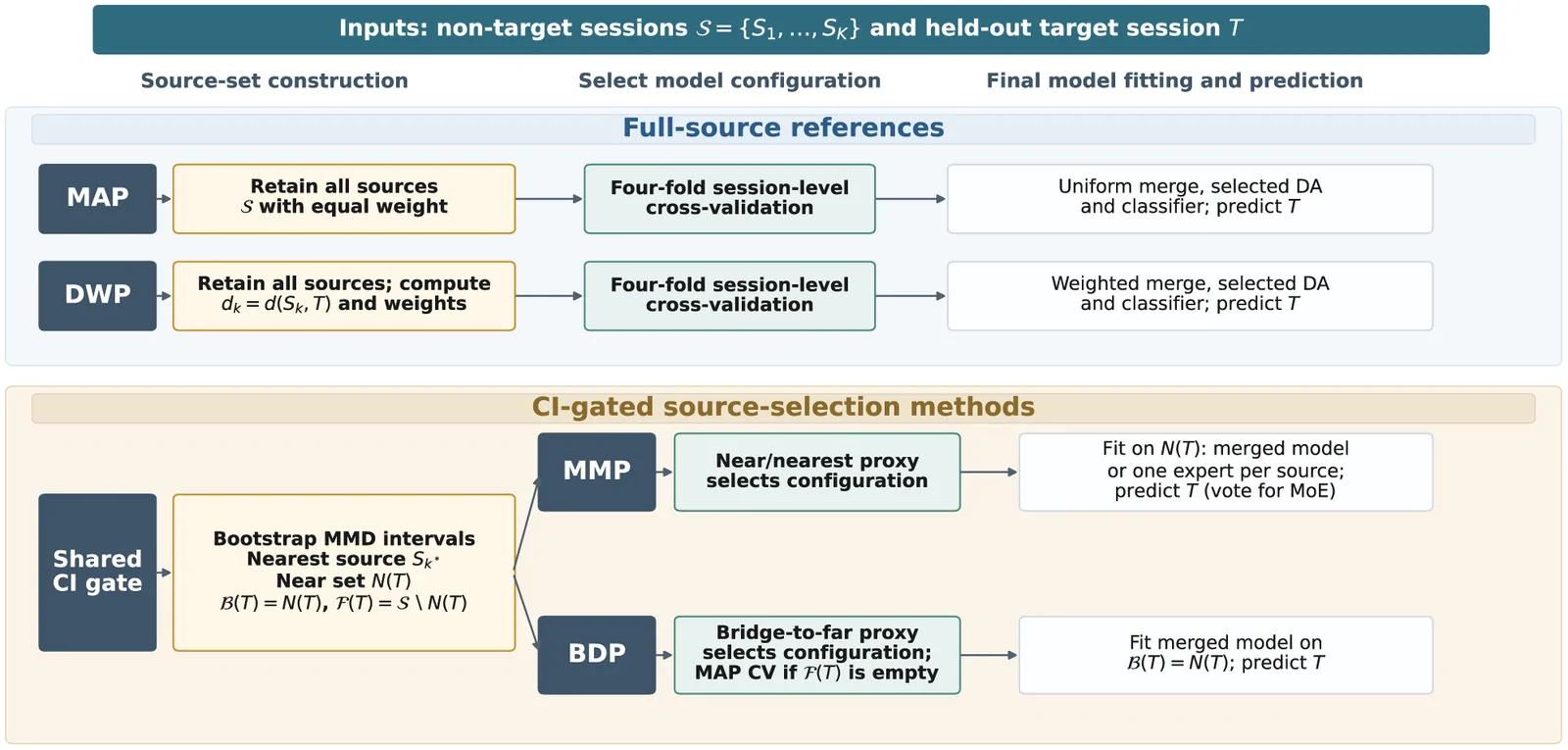
Conceptual overview of the four pipeline families. In summary, MAP pools all available source sessions uniformly; DWP reweights all sources according to their source-to-target MMD distances; and MMP and BDP share the CI-gated near set N(T)=B(T). MMP uses a near/nearest proxy task, whereas BDP leverages B(T) and F(T) for bridge-to-far proxy scoring. Crucially, both pipelines rely exclusively on *N*(*T*) for final model training.

We evaluate four distinct pipeline architectures, including two algorithmic MMP variants, yielding a total of five evaluated pipelines. The reference baselines, MAP and DWP, utilize all available source sessions without filtering. In contrast, MMP and BDP initially construct an identical, target-dependent source set partition, which they subsequently leverage through distinct computational mechanisms to select an optimal model configuration. Figure 1 illustrates these functional processing stages.

#### 2.2.1 MAP: merge-all-pool

MAP uses all available non-target sessions and serves as the simplest full-pooling baseline (Lotte et al., 2018). MAP employs session-level fourfold cross-validation over the non-target sessions to select a feature extraction, classifier architecture, and domain-adaptation combination. It retains the configuration achieving the highest cross-validated accuracy. MAP then merges all non-target sessions uniformly, applies the selected adaptation method, fits the selected classifier, and predicts the class labels for the held-out target session.

#### 2.2.2 DWP: distance-weighted pooling

DWP uses the identical fourfold configuration-selection procedures to MAP. After selecting a configuration, it computes the distribution distance from each source session to the target and assigns inverse-distance weights for final model training. Let *d*_*k*_ = *d*(*S*_*k*_, *T*) be the distance from source session *S*_*k*_ to target session *T* in the selected feature representation. Its normalized weight is given by $w_{k}={\left(d_{k}+\epsilon \right)}^{-1}/\underset{j}{\sum }{\left(d_{j}+\epsilon \right)}^{-1}$, where ϵ>0 prevents division by zero. DWP estimates *d*_*k*_ directly, without bootstrap averaging. The default distance metric is maximum mean discrepancy (MMD), which compares the empirical distributions of two samples through kernel-based similarities (Gretton et al., 2012).

Weighted pooling is implemented via deterministic sample replication with a scaling factor of 50. Each source session is replicated in proportion to its normalized weight, rounded to an integer number of copies, with at least one copy retained. The replicated sessions are concatenated into a single composite training set. DWP therefore retains every source session but gives greater influence to sessions exhibiting smaller estimated distributional distances.

#### 2.2.3 MMP: minimum-distance multi-source pipeline

MMP applies the CI-gated source-selection rule defined in Section 2.3. For target session *T*, this rule returns a near set *N*(*T*) and identifies the nearest source index k^*^. The corresponding retained session is $S_{k^{*}}$. MMP does not impose an additional fixed-size limit on *N*(*T*).

MMP selects a configuration via a near/nearest proxy task. If |*N*(*T*)|≥2, it treats $S_{k^{*}}$ as a proxy target and uses $N\left(T\right)\backslash \left\{S_{k^{*}}\right\}$ as proxy sources. If |*N*(*T*)| = 1, it uses the second-nearest session to *T* as the proxy source and keeps $S_{k^{*}}$ as the proxy target. This second-nearest session is leveraged only for configuration selection; final model training remains restricted to *N*(*T*). MMP never falls back to MAP cross-validation.

The two MMP variants share an identical near set and proxy task, but evaluate candidate configurations using distinct final combination strategies. The merge-then-adapt variant, MMP_mta_, concatenates the sessions in *N*(*T*) into a single training set before adaptation and classification. In contrast, the mixture-of-experts variant, MMP_moe_, trains one expert per near session, adapts each expert separately, and combines their predictions by a distance-weighted vote. The weights are fixed by distance rather than learned from target labels. When |*N*(*T*)| = 1, MMP_moe_ naturally reduces to a single expert model.

#### 2.2.4 BDP: bridge-domain pipeline

BDP operates on the same CI-gated source-set partition as MMP. Specifically, it defines the bridge set as B(T)=N(T) and the far set as F(T)=S\B(T). The bridge set is used exclusively for final model training. The far set is leveraged only to construct a proxy task for hyperparameter and model configuration selection.

When the far set F(T) is nonempty, BDP evaluates each candidate configuration by training a model on B(T) and testing its performance on F(T). If F(T) is empty, BDP reverts to the MAP cross-validation score over B(T) to select a configuration. This fallback strategy modifies only the configuration selecting scoring; final training remains restricted to B(T)=N(T). The selected configuration is then fitted on B(T) and evaluated on the held-out target session *T*. We also evaluated the reverse-direction Far → Bridge proxy task. Because its endpoint and source-selection summaries were nearly identical to the forward configuration, the main analysis reports only the Bridge → Far designation, labeled simply as BDP.

### 2.3 CI-gated source selection

MMP and BDP share a CI-gated source-selection rule. Here, source selection refers to the construction of a target-dependent near set and its far-set complement, while configuration selection and final model training are performed downstream. For each source session *S*_*k*_, we estimate the MMD distance *d*_*k*_ = *d*(*S*_*k*_, *T*) to the target session *T*. Subject-level bootstrap resampling gives the 95% confidence interval *C*_*k*_ = [*L*_*k*_, *U*_*k*_], evaluated at significance level α = 0.05. The nearest source session index is defined as $k^{*}=\arg\underset{k}{\min}d_{k}$. The near set is formally specified as:

$$N\left(T\right)=\left\{S_{k}:C_{k}\cap C_{k^{*}}\neq \emptyset \right\}.$$

Thus, a source session is retained when its distance confidence interval overlaps with that of the nearest source session. Here, CI overlap serves as a practical screening rule, not as a formal pairwise test; overlap of marginal confidence intervals does not generally establish whether two statistical estimates differ significantly (Schenker and Gentleman, 2001).

The CI-gating procedure employed *B* = 200 non-parametric bootstrap replicates. Within each bootstrap replicate, trials from the source and target sessions were resampled independently with replacement, preserving their original sample sizes. The confidence interval bounds (*L*_*k*_ and *U*_*k*_) were defined as the 2.5th and 97.5th percentiles of the resulting bootstrap distance distribution, with the point estimate *d*_*k*_ calculated as the empirical mean. Distances were computed as biased empirical squared MMD values using a Gaussian radial basis function (RBF) kernel with a fixed bandwidth parameter of σ = 1.0. The feature extraction pipeline was fitted exclusively on source-session data, and MMD was subsequently computed within the extracted candidate feature space. Consequently, the membership of *N*(*T*) varied across feature families and target folds. Importantly, classifier architecture and domain-adaptation algorithm choices were excluded from the distance calculation.

MMD was chosen because it compares entire empirical distributions rather than merely their means. When paired with an RBF kernel, MMD captures higher-order distributional differences beyond simple location shifts without requiring covariance-matrix inversion (Gretton et al., 2012). In contrast, Euclidean distance between session means captures location shifts, while Mahalanobis distance requires a stable, well-conditioned covariance matrix estimate. Furthermore, repeated Wasserstein-distance estimation would introduce prohibitive computational overhead within the bootstrap resampling loop. Although MMD is not uniquely optimal and its value remains sensitive to kernel scale, the fixed bandwidth used here serves as an operational implementation setting rather than a universal constant (Schrab et al., 2023).

We examined the sensitivity of CI coverage and RBF-kernel bandwidth parameters in separate single-factor sensitivity analyses. The tested conditions, aggregation protocols, and results are detailed in Section S1 of the Supplementary material.

In summary, the generated confidence intervals structure the source-session retention partition rather than serving as formal hypothesis tests for inter-session equality. MMP uses *N*(*T*) to construct its near/nearest proxy task, whereas BDP designates this same set as B(T) and leverages its complement F(T) for bridge-to-far proxy evaluation.

### 2.4 Feature extraction

We compared three distinct feature representations to ensure that the pipeline performance comparisons remained independent of any single feature family.

*Common Spatial Patterns (CSP)*. CSP produced log-variance features from the top eight spatially filtered components (bounded by the data rank and channel count) (Ramoser et al., 2000; Blankertz et al., 2008; Samek et al., 2014). These spatial filters maximize variance contrast between the two MI classes and were estimated exclusively from source-session labels only; target-session labels were never used during feature extraction.

*Raw log-variance (logvar)*. Channel-wise log-variance yielded three feature dimensions on BNCI2014-004 and 62 on Stieger2021. This representation summarizes band power directly without learning spatial projections.

*Tangent-space (TS) features*. Trial covariance matrices were projected onto the tangent space of the manifold of symmetric positive-definite (SPD) matrices (Barachant et al., 2012; Congedo et al., 2017). This produced six features on BNCI2014-004 and 1953 features on Stieger2021.

### 2.5 Classification

We employed linear discriminant analysis (LDA) with automatic shrinkage and a support vector machine with a radial basis function kernel (RBF-SVM) with *C* = 0.1 and gamma=scale. The SVM was a classifier option shared by every pipeline, rather than a separately optimized pipeline baseline. Its hyperparameters were held constant throughout the main benchmark to maintain a uniform configuration grid across all pipelines. A separate sensitivity analysis on BNCI2014-004 varied *C*∈{0.01, 0.1, 1, 10} and set gamma to either scale or auto without expanding the hyperparameter grid.

### 2.6 Domain adaptation

We evaluated four DA options: no adaptation, Subspace Alignment (SA) (Fernando et al., 2013) with a nominal maximum subspace dimension bound of *k*_max_ = 10, a Riemannian parallel-transport/Procrustes-style alignment method (PT), and Correlation Alignment (CORAL) (Sun et al., 2016). Across all three adaptation methods, target labels were strictly excluded; only unlabeled target samples were leveraged to estimate the alignment. Brief descriptions of each DA method are provided below.

*No adaptation*. The classifier was trained directly on the source-derived feature representation and applied to the target session without an alignment step. However, performance differences across pipelines could still arise from variations in source selection, sample weighting, model aggregation, or configuration selection.

*Subspace Alignment (SA)*. SA projects source and target features into low-dimensional subspaces and aligns the source basis with the target basis (Fernando et al., 2013). The nominal subspace dimension upper bound was set to *k*_max_ = 10. The effective dimension used in a fold was defined as *k*_eff_ = min(10, *r*_*S*_, *r*_*T*_), where *r*_*S*_ and *r*_*T*_ denote the source and target feature dimensionalities, respectively. This adaptive rule allowed a uniform upper bound to be applied across both low- and high-dimensional feature representations.

*Parallel-transport/Procrustes alignment (PT)*. PT aligns source representations with the target domain via a parallel-transport/Procrustes-style transformation (Rodrigues et al., 2019). Unlike SA, it operates directly on the geometric manifold of the feature representation rather than aligning two Euclidean subspace bases.

*CORAL*. CORAL matches the second-order statistics of the source and target distributions by aligning their covariance matrices (Sun et al., 2016). Specifically, it whitens the source feature distribution and recolors it using the covariance matrix estimated from the unlabeled target session. The classifier is then trained on the transformed source-domain features.

### 2.7 Datasets

We evaluated the pipelines on two public motor imagery EEG datasets accessed via MOABB (Jayaram and Barachant, 2018). Both tasks involved binary classification of left-hand versus right-hand motor imagery. Each task therefore had two classes and a chance accuracy of 50%. Table 1 summarizes the key characteristics of these datasets. The Stieger2021 (Stieger et al., 2021) dataset served as the primary dataset. It contains 62 subjects, between 7 and 11 recording sessions per subject, and 62 EEG channels sampled at 1 kHz. Across this dataset, the mean number of available source sessions per leave-one-session-out fold was 9.02. The BNCI2014-004 dataset served as a secondary, low-channel replication benchmark. It consists of 9 subjects, 5 sessions recorded on separate days, and 3 EEG channels (C3, Cz, C4) sampled at 250 Hz. For both datasets, continuous EEG signals were bandpass-filtered to 8–35 Hz and segmented into trial epochs prior to feature extraction.

**Table 1:** Dataset overview.

Dataset	Subjects	MI classes	Sessions per subject	Channels	Sampling rate (Hz)
Stieger2021	62	2 (center/right hand)	7–11	62	1,000
BNCI2014-004	9	2 (center/right hand)	5	3	250

### 2.8 Evaluation protocol

We employed a within-subject, leave-one-session-out evaluation scheme. Each session served sequentially as the unlabeled target domain, while the remaining sessions formed the source pool. All five pipelines were evaluated across identical subjects, target folds, and a full factorial grid comprising 3 feature representations × 2 classifiers × 4 domain-adaptation options, yielding |C|=24 candidate configurations. In total, this resulted in 71,520 configuration-fold evaluations on Stieger2021 and 5400 on BNCI2014-004.

For subject *s*, pipeline *p*, target fold *t*, and configuration c∈C, let *a*_*spt*_(*c*) denote the final decoding accuracy on the held-out target session. If subject *s* contains *J*_*s*_ target sessions, its fixed-configuration accuracy is defined as the unweighted mean across target folds,

$$a\left(s,p,c\right)=\frac{1}{J_{s}}\overset{J_{s}}{\underset{t=1}{\sum }}a_{spt}\left(c\right).$$

The best-configuration and mean-configuration summaries are

$$\text{Acc}max\left(s,p\right)=\underset{c\in C}{\max}a\left(s,p,c\right),\text{ Acc}mean\left(s,p\right)=\frac{1}{\left|C\right|}\underset{c\in C}{\sum }a\left(s,p,c\right).$$

Within each held-out target fold *t*, each pipeline assigns a proxy score *q*_*spt*_(*c*) to all 24 configurations without accessing labels from that target fold (refer to Section 2.2 for details). The highest-scoring configuration is then selected separately for that fold:

$${ĉ}_{spt}=\arg\underset{c\in C}{\max}q_{spt}\left(c\right),$$

and selected-configuration accuracy is

$$\text{Acc}auto\left(s,p\right)=\frac{1}{J_{s}}\overset{J_{s}}{\underset{t=1}{\sum }}a_{spt}\left({ĉ}_{spt}\right).$$

Different target folds may therefore select different model configurations. The subject-level Acc*auto* is the unweighted mean of the target decoding accuracies obtained from these fold-specific selections. For MAP and DWP, *q*_*spt*_(*c*) is the fourfold session-level cross-validation score. For MMP, it is the near/nearest proxy accuracy. For BDP, it is the Bridge → Far proxy accuracy; when F(T) is empty, BDP reverts to the MAP cross-validation score. Ties in proxy score are resolved using the deterministic ordering of the common configuration grid.

These summaries answer distinct research questions. Acc*max* reports the best achievable performance under ideal configuration choice, whereas Acc*mean* averages performance across the complete fixed grid. Crucially, Acc*auto* evaluates the configuration selected by the pipeline's own proxy score. Unlike Acc*max* and Acc*mean*, Acc*auto* evaluates source retention, configuration selection, and final model fitting together as a complete pipeline; it is not used to attribute performance to source retention alone. Keeping these three summaries separate prevents over-interpretation of the post-hoc configurations (Cawley and Talbot, 2010; Varoquaux et al., 2017). We also report the best-to-mean gap Acc*max*(*s, p*)−Acc*mean*(*s, p*), source-session utilization, DA gains, and execution time. For DWP, the effective number of weighted sessions is defined as $n_{\text{eff}}={\left(\underset{k}{\sum }w_{k}^{2}\right)}^{-1}$. When subject and pipeline indices are omitted for brevity, we write Acc*max*, Acc*mean*, and Acc*auto*.

Friedman tests were conducted to compare all five pipelines within each dataset and performance summary. Pairwise Wilcoxon signed-rank tests serve as nominal follow-up contrasts. They describe the direction of matched subject-level differences but do not establish a familywise-confirmed ranking. This analysis follows the general principle that learning procedures should be compared on matched evaluation units (Demšar, 2006).

## 3 Results

Stieger2021 provides the primary statistical evidence, while BNCI2014-004 serves as a smaller replication dataset. Table 2 reports Acc*auto*, Acc*max*, and Acc*mean* as percentages (mean ± SD across matched subjects). MMP_mta_ and BDP share an identical final evaluation matrix: for every target fold and fixed configuration, both pipelines employ the same feature-specific CI-gated source set and the same final model-fitting procedure. Their fixed-configuration summaries are therefore identical by construction. In contrast, their Acc*auto* values diverge because MMP and BDP apply different proxy scoring mechanisms for data-driven configuration selection.

**Table 2:** Cross-dataset evaluation summary.

Dataset	Pipeline	Subjects	Acc*auto*	Acc*max*	Acc*mean*
Stieger2021	MAP	62	64.56 ± 10.83	71.73 ± 10.04	60.12 ± 6.15
Stieger2021	DWP	62	64.60 ± 10.93	71.48 ± 9.85	61.13 ± 6.28
Stieger2021	MMP_mta_	62	62.45 ± 10.31	71.38 ± 9.96	59.60 ± 5.96
Stieger2021	MMP_moe_	62	62.49 ± 10.44	69.38 ± 10.30	57.30 ± 5.08
Stieger2021	BDP	62	64.62 ± 10.85	71.38 ± 9.96	59.60 ± 5.96
BNCI2014-004	MAP	9	71.20 ± 10.22	75.12 ± 11.05	68.80 ± 9.63
BNCI2014-004	DWP	9	71.27 ± 10.19	74.74 ± 11.17	68.72 ± 9.64
BNCI2014-004	MMP_mta_	9	74.17 ± 11.51	74.40 ± 11.31	68.03 ± 9.75
BNCI2014-004	MMP_moe_	9	69.64 ± 10.41	74.41 ± 11.20	67.50 ± 9.48
BNCI2014-004	BDP	9	73.48 ± 11.18	74.40 ± 11.31	68.03 ± 9.75

Acc*auto*, Acc*max*, and Acc*mean* are reported as percentages (mean ± SD across matched subjects).

**Figure 2:**
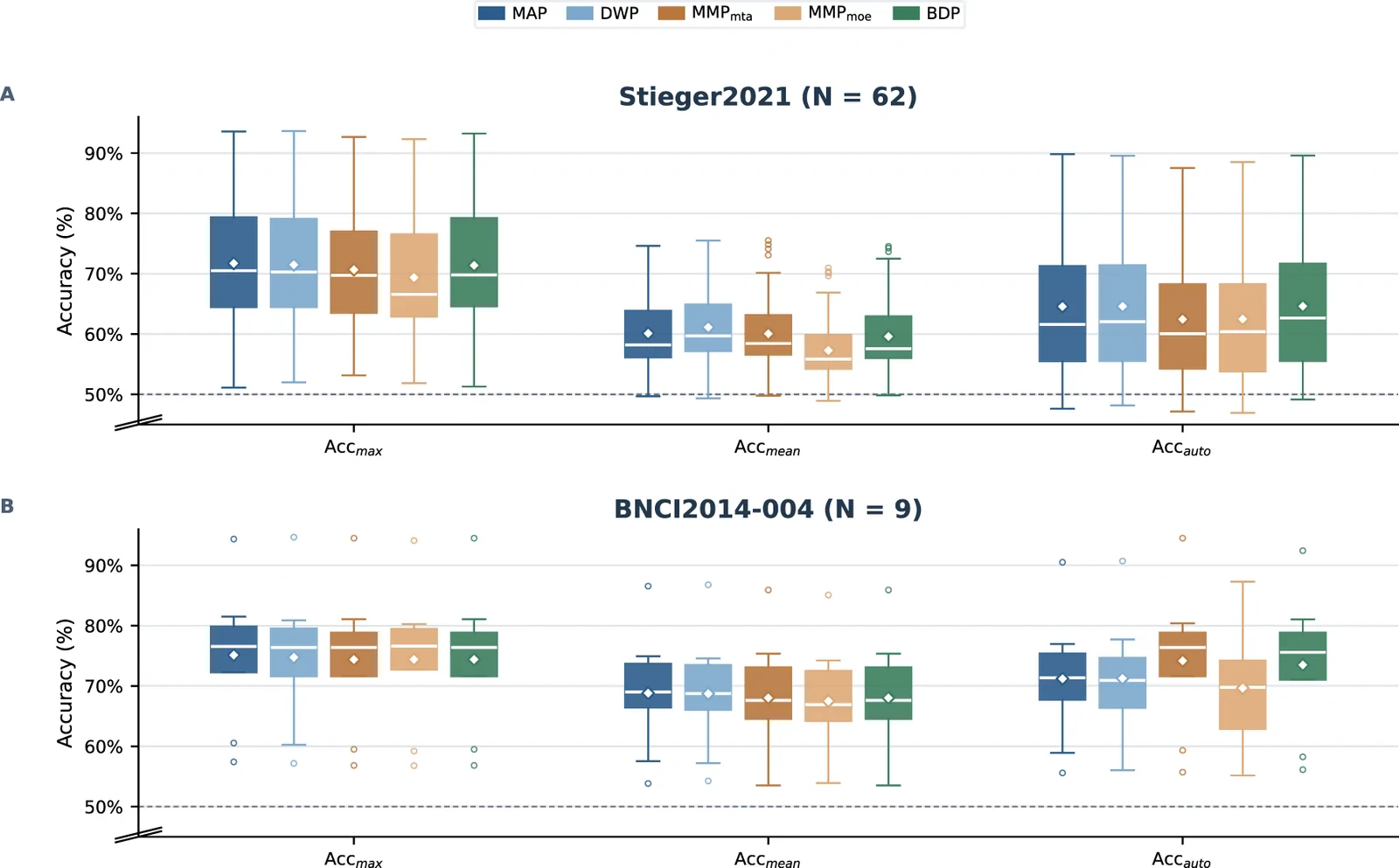
Grouped boxplot comparison of MAP, DWP, MMP_mta, MMP_moe, and BDP for Acc_*max*_, Acc_*mean*_, and Acc_*auto*_ on **(A)** Stieger2021 and **(B)** BNCI2014-004. Diamonds mark the means, and the dashed line marks 50% chance accuracy.

Figure 2 provides a grouped comparison of Acc*max*, Acc*mean*, and Acc*auto* across pipelines for each dataset.

### 3.1 Best-configuration accuracy and best-to-mean gap

**Figure 3:**
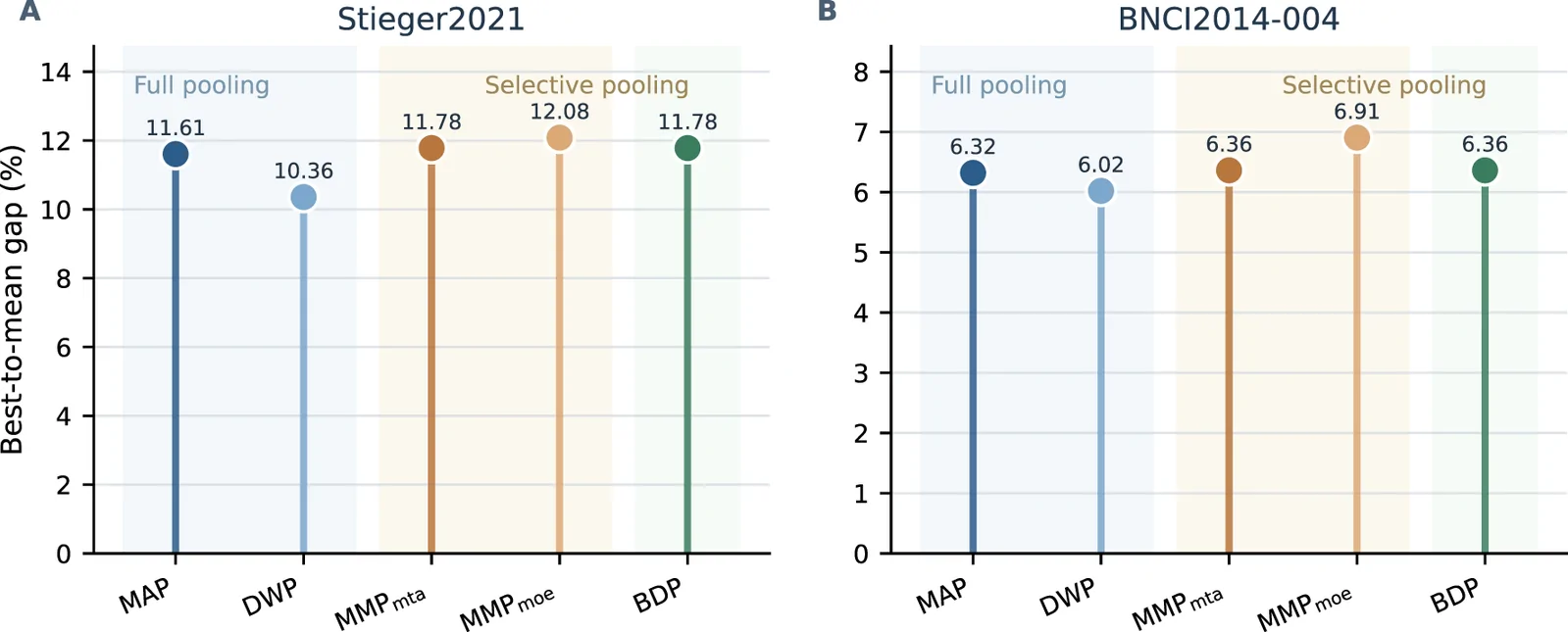
Best-to-mean gap across MAP, DWP, MMP_*mta*_, MMP_*moe*_, and BDP on **(A)** Stieger2021 and **(B)** BNCI2014-004. The gap is defined as Acc*max*−Acc*mean* and reported as a percentage. A larger value denotes a larger difference between the best and average fixed-configuration results.

Best-configuration accuracy Acc*max* represents the subject-level maximum achieved over the fixed grid. The best-to-mean gap Acc*max*−Acc*mean* measures the performance gain of this maximum relative to the overall mean across the grid. On Stieger2021, Acc*max* was 71.73% for MAP, 71.48% for DWP, 71.38% for both MMP_mta_ and BDP, and 69.38% for MMP_moe_ (Table 2; Figure 2). The corresponding best-to-mean gaps were 10.36% for DWP, 11.61% for MAP, 11.78% for MMP_mta_ and BDP, and 12.08% for MMP_moe_ (Figure 3). As noted, MMP_mta_ and BDP exhibit identical fixed-configuration summaries because they share the same final evaluation matrix; however, this equality does not establish performance equivalence with full pooling. Because Acc*max* does not rely on a pipeline-specific proxy selector, the MAP–BDP comparison describes the fixed-grid consequence of replacing all-source training with CI-gated near-source training. Specifically, the near-source value was 0.35% lower than MAP on Stieger2021. Table 3 provides a complementary view by listing the two top-performing globally fixed configurations for each pipeline and dataset.

**Table 3:** Top two globally fixed configurations by pipeline and dataset.

Pipeline	Rank	Stieger2021		BNCI2014-004	
		**Configuration**	**Acc. (%)**	**Configuration**	**Acc. (%)**
MAP	1	TS/LDA/CORAL	69.43	CSP/SVM/PT	74.52
	2	TS/LDA/none	69.43	CSP/LDA/SA	74.43
DWP	1	CSP/LDA/SA	67.70	CSP/LDA/SA	74.22
	2	CSP/SVM/SA	67.37	CSP/LDA/PT	73.83
MMP_mta_	1	TS/LDA/CORAL	68.63	CSP/LDA/SA	73.92
	2	TS/LDA/none	68.59	CSP/LDA/PT	73.59
MMP_moe_	1	CSP/LDA/SA	67.22	CSP/LDA/SA	74.03
	2	CSP/LDA/PT	65.92	CSP/LDA/PT	73.72
BDP	1	TS/LDA/CORAL	68.63	CSP/LDA/SA	73.92
	2	TS/LDA/none	68.59	CSP/LDA/PT	73.59

Configurations are shown as feature/classifier/domain-adaptation triples. For each pipeline, each fixed configuration is first averaged across target folds within each subject and then averaged across subjects to rank the resulting global averages. Table 2 instead first computes the mean accuracy for each configuration across target folds within each subject, extracts the maximum accuracy within each subject, and finally averages these subject-specific maxima across subjects; consequently, the two aggregation sequences do not necessarily yield the same numerical value. Accuracy values are expressed as percentages. Throughout, BDP denotes the Bridge → Far proxy variant retained for the main analysis.

On BNCI2014-004, MAP achieved the highest Acc*max* (75.12%), while the shared MMP_mta_/BDP near-source value was 74.40%, or 0.72% lower. The five best-to-mean gaps ranged from 6.02% to 6.91%.

Table 3 demonstrates that the leading fixed configurations differed by dataset. TS/LDA configurations ranked first for MAP, MMP_mta_, and BDP on Stieger2021. CSP configurations occupied all first-ranked positions on BNCI2014-004. MMP_mta_ and BDP exhibited identical leading configurations because they shared the same final evaluation matrix. In contrast, DWP and MMP_moe_ instead ranked CSP-based configurations first on Stieger2021. These global rankings do not coincide with Acc*max*, which averages each configuration across target folds before taking a separate maximum within each subject. They are also distinct from the subject- and fold-specific data-driven selections summarized by Acc*auto*.

### 3.2 Mean accuracy across configurations

On Stieger2021, DWP achieved the highest configuration-averaged accuracy Acc*mean* value at 61.13%, followed by MAP at 60.12%, MMP_mta_ and BDP at 59.60%, and MMP_moe_ at 57.30% (Figure 2). The MAP–BDP difference was 0.52%. Like Acc*max*, this contrast evaluates performance across the complete fixed grid without involving the BDP proxy selector. Representative paired subject-level tests are summarized in Table 4. In the reported nominal contrasts, DWP significantly outperformed MAP, MMP_mta_, BDP, and MMP_moe_ (all *p* < 0.001). Similarly, MAP maintained a significant advantage over MMP_mta_, BDP, and MMP_moe_ (all *p* < 0.001).

**Table 4:** Representative paired subject-level comparisons on Acc*mean*.

**Dataset**	Comparison	W/T/L	Mean Δ (%)	Median Δ (%)	*p*
Stieger2021	MAP vs. DWP	6/0/56	−1.01	−1.09	< 0.001
Stieger2021	MAP vs. MMP_mta_	49/0/13	0.52	0.39	< 0.001
Stieger2021	MAP vs. MMP_moe_	60/0/2	2.82	2.55	< 0.001
Stieger2021	MAP vs. BDP	49/0/13	0.52	0.39	< 0.001
Stieger2021	DWP vs. BDP	59/0/3	1.53	1.46	< 0.001
BNCI2014-004	MAP vs. DWP	6/0/3	0.08	0.19	0.652
BNCI2014-004	MAP vs. MMP_mta_	8/0/1	0.77	0.63	0.012
BNCI2014-004	MAP vs. MMP_moe_	8/0/1	1.30	1.46	0.008
BNCI2014-004	MAP vs. BDP	8/0/1	0.77	0.63	0.012
BNCI2014-004	DWP vs. BDP	8/0/1	0.69	0.86	0.027

W/T/L counts wins, ties, and losses for the first-named pipeline; Mean Δ and Median Δ are first-named minus second-named mean accuracy across configurations, reported as percentage differences.

On BNCI2014-004, MAP and DWP exhibited comparable performance (68.80% and 68.72%, respectively), with MMP_mta_ and BDP matching at 68.03% and MMP_moe_ at 67.50%. The MAP–BDP difference was 0.77%. MAP did not differ significantly from DWP (*p* = 0.652), but it exceeded MMP_mta_ (*p* = 0.012), MMP_moe_ (*p* = 0.008), and BDP (*p* = 0.012). DWP likewise outperformed MMP_mta_ (*p* = 0.027), MMP_moe_ (*p* = 0.004), and BDP (*p* = 0.027). These nominal contrasts warrant cautious interpretation because BNCI2014-004 comprises a small matched-subject sample and (*N* = 9) displays a compressed performance spread.

**Figure 4:**
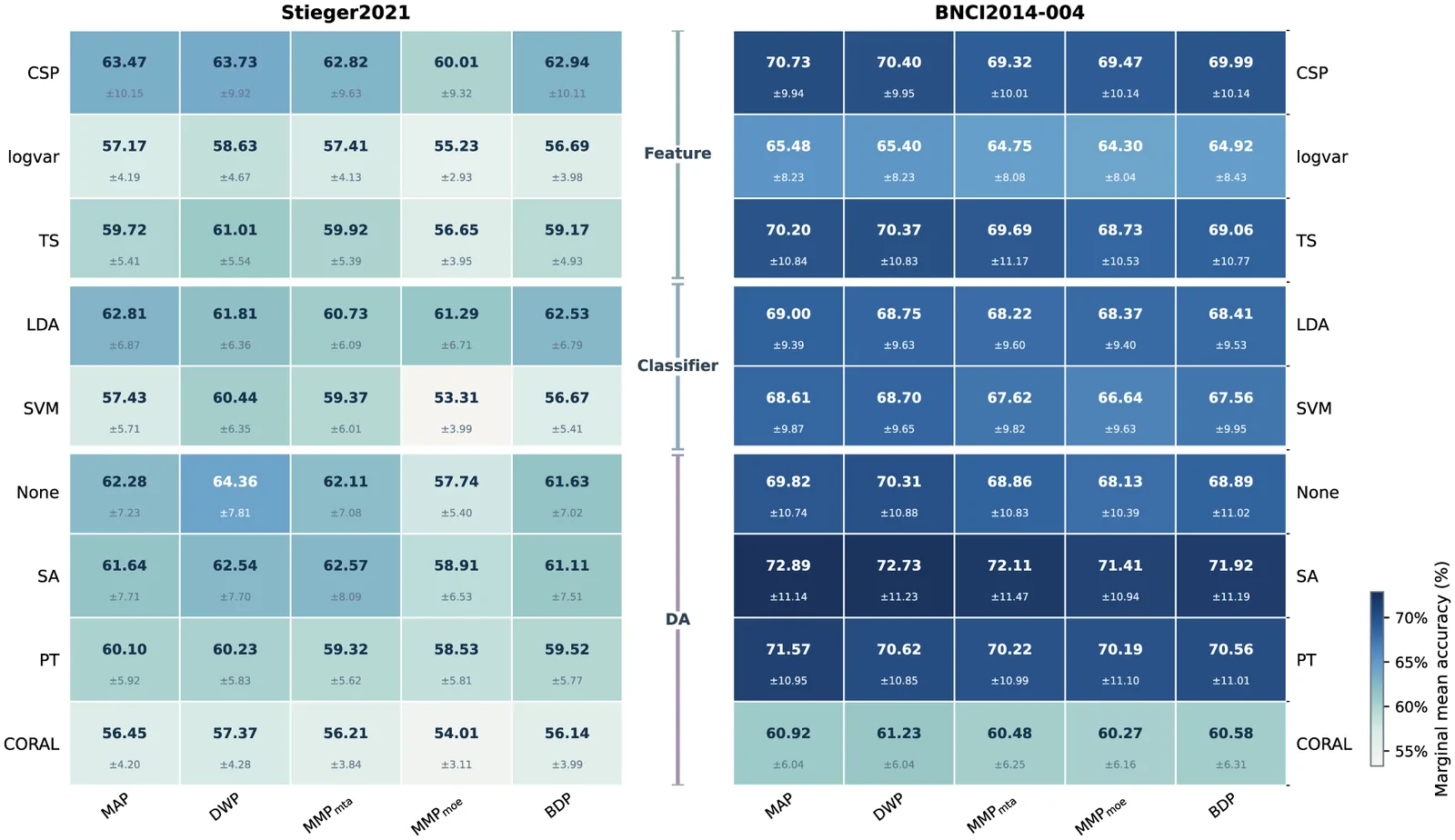
Pipeline-specific marginal accuracy means for configuration-averaged decoding accuracy. Within each pipeline and subject, the feature extraction rows average over all classifier architectures and DA choices, the classifier rows average over all feature families and DA choices, and the DA rows average over all feature families and classifier architectures. Cell annotations report the subject-level mean and standard deviation for each marginal summary, both expressed as percentages.

Figure 4 presents the accuracy averaged separately over feature extraction, classifier architecture, and domain-adaptation choices. CSP attained the highest feature-marginal mean for all five pipelines across both datasets. This finding contrasts with Table 3, where the top-performing individual Stieger2021 configurations for MAP, MMP_mta_, and BDP utilized TS in conjunction with LDA. Consequently, a feature family can yield therefore have the top-performing marginal mean without appearing in the highest specific feature–classifier–adaptation combination. Classifier and domain-adaptation marginals exhibited less consistent variation across pipelines.

### 3.3 Performance for data-driven configuration selection

We next examined selected-configuration accuracy Acc*auto*, the fold-averaged decoding accuracy obtained after each pipeline applied its own proxy score to select a feature, classifier, and domain-adaptation option. This summary evaluates each complete pipeline; it is not used to estimate the effect of source retention alone. On Stieger2021, BDP, DWP, and MAP attained three highest mean Acc*auto* values, ranging from 64.56% to 64.62%. In comparison, MMP_moe_ and MMP_mta_ yielded means of 62.49% and 62.45%. respectively (Figure 2).

On BNCI2014-004, selected-configuration accuracy was highest for MMP_mta_ at 74.17%, followed by BDP at 73.48%, DWP at 71.27%, MAP at 71.20%, and MMP_moe_ at 69.64%. The full five-pipeline performance spread was 4.53%.

MMP_mta_ and BDP provide a direct comparison of configuration proxy scoring rules because they share the same near-source final evaluation matrix. On Stieger2021, MMP_mta_ was 2.17% lower than BDP (*p* < 0.001). On BNCI2014-004, it was 0.69% higher, although this difference did not reach statistical significant (*p* = 0.156). The relative performance of the two proxy rules was therefore dataset-dependent.

Table 5 reports the paired statistical data-driven comparisons after configuration selection. On Stieger2021, MAP significantly differ from DWP (W/T/L = 27/1/34, *p* = 0.422) or BDP (27/0/35, *p* = 0.301) in the reported nominal contrasts. However, MAP significantly exceeded both MMP variants (51/0/11 and 48/0/14, both *p* < 0.001). On BNCI2014-004, MAP significantly lower than MMP_mta_ (0/0/9, *p* = 0.004) and BDP (1/0/8, *p* = 0.027). It did not differ significantly from DWP and statistically exceeded MMP_moe_ (7/0/2, *p* = 0.039). The Stieger2021 pipeline ranking was therefore not replicated on BNCI2014-004. Interpretation of the latter dataset is limited by its nine-subject sample size (*N* = 9) and small performance differences across pipeline means.

**Table 5:** Representative paired subject-level comparisons for Acc*auto*.

Dataset	Comparison	W/T/L	Mean Δ (%)	Median Δ (%)	*p*
Stieger2021	MAP vs. DWP	27/1/34	−0.04	−0.13	0.4221
Stieger2021	MAP vs. MMP_mta_	51/0/11	2.11	1.60	< 0.0001
Stieger2021	MAP vs. MMP_moe_	48/0/14	2.07	1.55	< 0.0001
Stieger2021	MAP vs. BDP	27/0/35	−0.06	−0.25	0.3011
Stieger2021	MMP_mta_ vs. BDP	8/0/54	−2.17	−1.62	< 0.0001
BNCI2014-004	MAP vs. DWP	4/0/5	−0.07	−0.20	0.7344
BNCI2014-004	MAP vs. MMP_mta_	0/0/9	−2.97	−3.00	0.0039
BNCI2014-004	MAP vs. MMP_moe_	7/0/2	1.56	1.55	0.0391
BNCI2014-004	MAP vs. BDP	1/0/8	−2.28	−1.92	0.0273
BNCI2014-004	MMP_mta_ vs. BDP	4/3/2	0.69	0.00	0.1563

W/T/L counts wins, ties, and losses for the first-named pipeline; Mean Δ and Median Δ are first-named minus second-named auto-selected performance, reported as percentage differences.

### 3.4 Omnibus subject-level statistical comparison

Table 6 presents the omnibus five-pipeline Friedman test results. All three Stieger2021 tests were statistically significant. The test statistics were χ^2^(4) = 58.55 for Acc*max*, 177.42 for Acc*mean*, and 86.41 for Acc*auto* (all *p* < 0.001). Kendall's concordance coefficient *W* was 0.236, 0.715, and 0.348, respectively. The effect size and between-pipeline variance occurred for configuration-averaged accuracy Acc*mean*.

**Table 6:** Subject-level omnibus Friedman tests across the five main pipelines for the three main performance summaries.

Dataset	Summary	Subjects	χ^2^(4)	*p*	Kendall's *W*
Stieger2021	Acc*max*	62	58.5502	< 0.0001	0.2361
Stieger2021	Acc*mean*	62	177.4194	< 0.0001	0.7154
Stieger2021	Acc*auto*	62	86.4129	< 0.0001	0.3484
BNCI2014-004	Acc*max*	9	9.4269	0.0513	0.2619
BNCI2014-004	Acc*mean*	9	23.0877	0.0001	0.6413
BNCI2014-004	Acc*auto*	9	24.0226	< 0.0001	0.6673

On BNCI2014-004, the omnibus tests were likewise significant for Acc*mean* (χ^2^(4) = 23.09, *p* < 0.001, *W* = 0.641) and Acc*auto* (χ^2^(4) = 24.02, *p* < 0.001, *W* = 0.667). The Acc*max* comparison did not reach the 0.05 threshold (χ^2^(4) = 9.43, *p* = 0.051, *W* = 0.262). Thus, inter-pipeline differences in the best achievable configuration (Acc*max*) were smaller than those observed in those observed in average performance (Acc*mean*) or data-driven, pipeline-selected configurations (Acc*auto*).

### 3.5 Source selection

**Figure 5:**
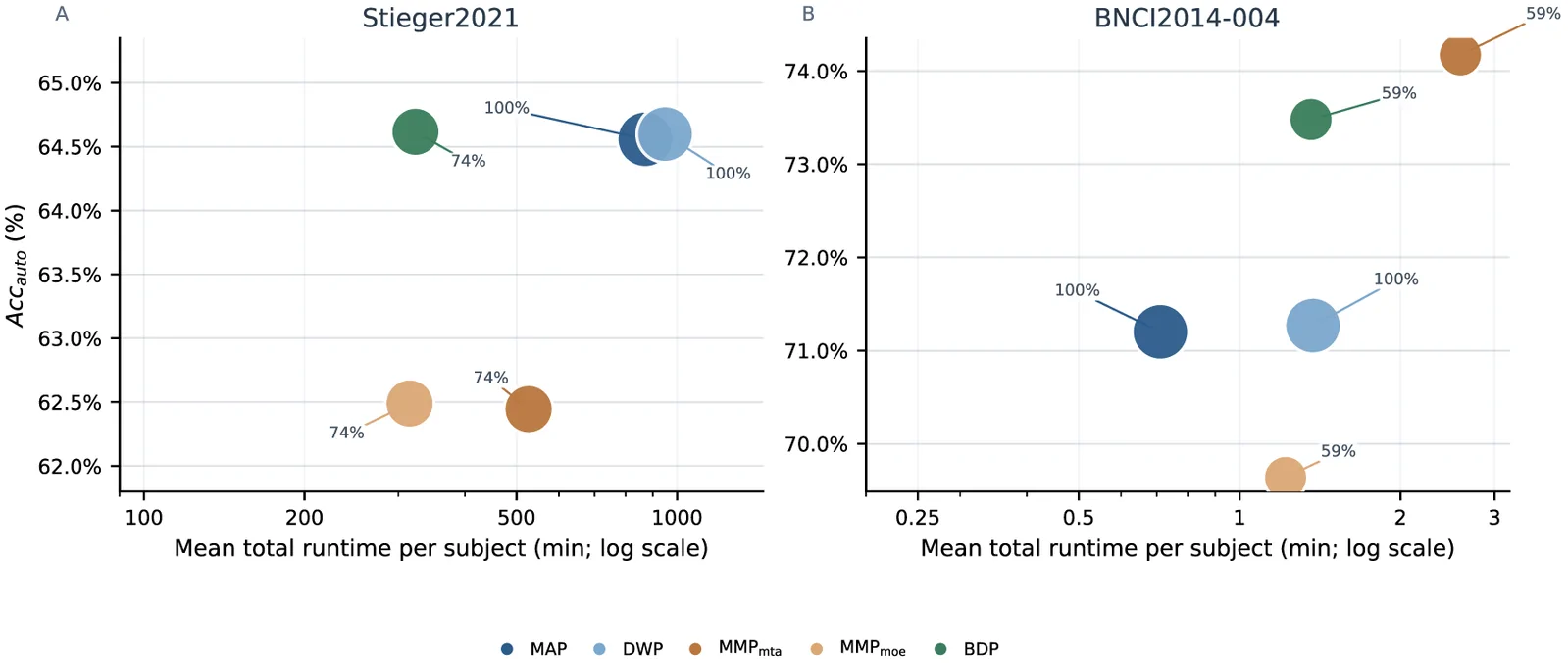
Combined view of runtime, performance, and source utilization for **(A)** Stieger2021 and **(B)** BNCI2014-004. Each point plots selected-configuration accuracy Acc*auto* against mean total runtime per subject on a log-scaled x-axis. Point size and the accompanying label report mean source utilization, defined as the percentage of available source sessions retained by the source rule.

Figure 5 and Table 7 report source-session retention and computational execution time. MAP and DWP retained all available source sessions without filtering. For MMP and BDP, the retained-session count and source utilization were computed before pipeline-specific configuration selection and averaged over subject–target-fold–feature gating combinations. Because both MMP variants and BDP utilize an identical CI-gated near set, they yield an identical gate-level retention summary. Under the default 95% CI, the mean retained-session count was 6.67 sessions, representing a mean utilization of 74.29% on Stieger2021; the corresponding values were 2.34 sessions and 58.52% on BNCI2014-004. Crucially, BDP's MAP-style boundary rule modifies only configuration scoring when the far set F(T) is empty; it does not alter *N*(*T*) or the set of sessions retained by the screening gate.

**Table 7:** Source retention and computational cost.

Dataset	Pipeline	Mean source-set size	Mean utilization (%)	Mean total runtime per subject (min)
Stieger2021	MAP	9.02	100.0	871.61
Stieger2021	DWP	9.02	100.0	949.66
Stieger2021	MMP_mta_	6.67	74.29	526.54
Stieger2021	MMP_moe_	6.67	74.29	314.96
Stieger2021	BDP	6.67	74.29	323.04
BNCI2014-004	MAP	4.00	100.0	0.71
BNCI2014-004	DWP	4.00	100.0	1.37
BNCI2014-004	MMP_mta_	2.34	58.52	2.59
BNCI2014-004	MMP_moe_	2.34	58.52	1.22
BNCI2014-004	BDP	2.34	58.52	1.36

For MMP and BDP, mean source-set size and utilization are gate-level averages over subject–target-fold–feature gates computed before pipeline-specific configuration selection. Counts and utilization percentages were averaged separately. MAP and DWP use all available non-target sessions. Runtime is the mean wall-clock time per subject over all target folds and 24 configurations.

### 3.6 Sensitivity analyses

Changing CI coverage influenced retained-source volume substantially more than fixed-configuration accuracy. On BNCI2014-004, gate-level source utilization decreased from 65.56% with under 99% coverage to 52.04% with under 90% coverage, while Acc*mean* ranged narrowly from 67.94% to 68.13% and Acc*max* ranged from 74.21% to 74.50%. On Stieger2021, utilization decreased from 80.24% to 69.71%, whereas Acc*mean* ranged from 60.44% to 60.75% and Acc*max* ranged from 72.29% to 72.50%. These descriptive results indicate that CI coverage effectively controlled retained-source volume over the tested range, though they do not identify an optimal confidence level.

The effect of kernel bandwidth varied by dataset. On BNCI2014-004, Acc*mean* ranged from 67.85% to 68.17% and Acc*max* from 74.16% to 74.39% across the six bandwidth conditions, whereas source utilization ranged from 46.30% to 63.15%. On Stieger2021, a fixed bandwidth of σ = 1.0 retained 74.29% of the available sources and produced Acc*mean* = 60.59% and Acc*max* = 72.40%. In contrast, adaptive settings retained only 28.39–42.32% of the sources and produced Acc*mean* values of 58.70–59.33% and Acc*max* values of 70.78–71.05%. Their mean gate Jaccard similarities relative to the fixed condition ranged from 0.342 to 0.548. Kernel scale therefore had a more pronounced impact on source-set composition and fixed-configuration accuracy on Stieger2021. Full protocols and condition-level summaries are provided in the Supplementary material.

In the BNCI2014-004 SVM sensitivity analysis, the total spread in Acc*max* was 0.21–0.26% across pipelines. In contrast, Acc*mean* varied by approximately 3.45% because it averages across all configurations, including suboptimal low-performing SVM settings. The default *C* = 0.1, gamma=scale configuration attained the highest MAP Acc*max* and remained within 0.13% of the highest Acc*max* for MMP_mta_ and BDP. The complete fixed-configuration results are reported in the Supplementary material.

### 3.7 Runtime and efficiency

Figure 5 and Table 7 compare selected-configuration accuracy and runtime with gate-level source retention.

Computational execution time was measured on an Ubuntu workstation equipped with an Intel Core i9-14900K CPU and 128 GB RAM, leveraging subject-level parallel processing. Reported execution times encompass feature extraction, CI-gating or distance computation, configuration scoring, and final target prediction across all leave-one-session-out folds and all 24 candidate configurations. These exclude data downloading, preprocessing-cache construction, figure generation, and table rendering. These values reflect the total computational cost of evaluating the complete benchmark search space in the current implementation, rather than inference latency of deploying a single pre-selected model in deployment.

On Stieger2021, MAP, DWP, and BDP achieved comparable mean selected-configuration accuracies Acc*auto* values between 64.56% and 64.62%. Notably, BDP required only 37% of MAP's execution time, while the shared CI gate retained 74.29% of the available source sessions on average across target-fold–feature gating combinations. DWP attained a similar Acc*auto* score incurred higher computational overhead than MAP. MMP_moe_ executed slightly faster than BDP but yielded lower Acc*auto*; whereas MMP_mta_ was both computationally slower less accurate than BDP. The primary advantage of BDP on Stieger2021 is therefore one of computational efficiency, not an accuracy improvement over full pooling.

## 4 Discussion

### 4.1 Principal findings

No pipeline ranked first on every performance summary. Specifically, MAP achieved the best-configuration accuracy Acc*max*, whereas DWP attained the highest configuration-averaged accuracy Acc*mean*. On Stieger2021, MMP_mta_ and BDP were 0.35% below the leading Acc*max* and 1.53% below the leading Acc*mean*, whereas MMP_moe_ lagged by 2.35% and 3.83%, respectively. In contrast, on BNCI2014-004, all proposed pipelines were within 1.30% of the leading fixed-configuration benchmarks. This equality in fixed-configuration metrics is imposed by the design and does not establish that CI-gating matches full pooling. Instead, two pipelines differed in selected-configuration accuracy Acc*auto* because they employed distinct proxy scores to select a configuration. MMP_moe_, which used a fixed distance-weighted vote, had lower values on all three summaries. The main practical result of BDP was lower resource use: on Stieger2021 it matched the MAP/DWP Acc*auto* performance while using fewer source sessions and requiring significantly less computational execution time.

### 4.2 Why selection alone is not enough

The results do not indicate that retaining fewer source sessions automatically improves classification accuracy. Two sessions recorded from the same subject can exhibit global distributional shifts yet still contain valuable discriminative information for binary class separation. Consequently, inter-session distance alone is insufficient to determine whether incorporating a given session will facilitate positive transfer.

The CI-overlap gating rule determined which source sessions were admitted into the near proxy set *N*(*T*), with its primary observable effect being a reduction in retained session volume. The proxy selection rule subsequently leveraged this partitioned source set to evaluate and select a candidate configuration. BDP integrates these two mechanisms, yielding computational efficiency and decoding accuracy trade-offs reported in Stieger2021.

Both MMP and BDP initialize from the same CI-gated near set *N*(*T*) and employ that set for final fixed-configuration model fitting. Their fundamental divergence stems from configuration selection: MMP formulates a near/nearest proxy task, whereas BDP constructs a bridge-to-far proxy task.

The present results show that inter-session distance captures only a fraction of the domain transfer dynamics. More broadly, unsupervised model selection in the absence of target labels remains difficult (You et al., 2019). On Stieger2021, MMP_mta_ had lower Acc*auto* than MAP, DWP, and BDP. Because MMP_mta_ and BDP shared identical final model fits, their divergent Acc*auto* values outcomes originated entirely from the distinct configurations selected by their respective proxy scoring functions.

This distinction aligns with existing literature on negative transfer, which characterizes source relevance metrics as informative yet fallible proxies, and with evidence demonstrating that baseline alignment methods can achieve competitive performance when favorable data geometries exist (Zhang et al., 2023; He and Wu, 2025).

Furthermore, the inclusion of domain adaptation did not alter the macro-level ranking across pipelines. Its marginal results varied by pipeline and dataset, so our benchmark provides no evidence of a systematic interaction between feature adaptation and source selection strategies.

Our sensitivity analyses disentangle two distinct functional properties of the screening gate. CI coverage controlled the volume of retained source sessions, with little change in fixed-configuration accuracy over the tested spectrum. Kernel bandwidth had a larger effect on source-set composition and produced dataset-dependent accuracy changes. CI-gated selection should therefore be interpreted as a highly controllable, but non-scale-free, source-selection paradigm.

Finally, the fixed-grid MAP–BDP comparison and the matched MMP_mta_–BDP comparison address different questions. The former describes the consequence of replacing all-source fitting with CI-gated near-source fitting without invoking the BDP proxy selector. The latter compares two proxy rules on the same near-source final evaluation matrix. Neither comparison treats source retention and configuration selection as interchangeable modules. Instead, Acc*auto* evaluates how they operate together within each complete pipeline.

### 4.3 Interpretation of the performance summaries

The three summaries address different practical questions. Acc*max* asks what best-configuration accuracy achievable by each pipeline family under ideal tuning. Acc*mean* asks what mean accuracy the pipeline maintains when results are averaged over the full configuration space. Acc*auto* asks how the full pipeline behaves when it commits to one configuration by its own internal rule and applies that configuration to the held-out target session. The best-to-mean gap, defined as Acc*max*−Acc*mean*, bridges the first two by quantifying how much a pipeline benefits from favorable tuning relative to its average-case behavior. Although complementary, these summaries are related, but they are not interchangeable.

The literature has not converged on a single optimal cross-session strategy partly because different studies optimize optimize for disparate evaluation metrics. A method that excels under ideal tuning may not be preferred once it commits to a single operating configuration, while a method that is strong on average may not deliver the highest single-configuration ceiling. Consequently, deployment efficacy claims based on best-configuration accuracy alone can be misleading. Evaluating how historical source sessions are used should be interpreted together with how configurations are chosen, and the best-to-mean gap helps connect ceiling and average-case behavior.

### 4.4 Implications for method selection

The results point to different methodological choices depending on specific operating goals. MAP remains the reference benchmark when the best attainable ceiling accuracy is the main target, whereas DWP remains the reference when average performance across the full configuration space matters most. BDP is most advantageous when the goal is to keep selected-configuration accuracy close to MAP/DWP while substantially reducing computation cost: on Stieger2021 it stayed in the MAP/DWP selected-performance range while requiring only about 37% of MAP's execution time. This should be interpreted as an efficiency result, not as evidence that BDP outperforms the all-source methods in accuracy. MMP_mta_ remains analytically informative because it demonstrates that the CI-gated retained-source partition can preserve fixed-configuration performance, even though its near/nearest proxy rule was not the optimal choice at the selected operating point.

Methodologically, these results suggest that future performance gains are far more likely to come from improving configuration-selection rules on retained session sets than from increasingly aggressive source-session pruning alone.

This framing also distinguishes the present benchmark from rehabilitation-oriented recalibration studies, where the primary objective is the prospective reduction of user calibration burden rather than a controlled comparison of source-session organization rules (Giles et al., 2022).

### 4.5 Limitations

This study has several notable limitations. First, the benchmark evaluates only two MI-BCI datasets, so the conclusions cannot be assumed to extend automatically to other paradigms such as P300 or SSVEP, or to substantially different subject populations. Second, the task is binary motor imagery; multiclass decoding may alter both the geometry of session drift and the relative utility of selective domain transfer. Third, best-configuration Acc*max* is inherently optimistic because it assumes ideal configuration selection, whereas selected-configuration accuracy Acc*auto* remains a benchmark analog rather than a prospective, real-time deployment trial. Fourth, all analyses are conducted offline and employ fixed DA hyperparameters, leaving online recalibration and adaptive hyperparameter selection untested. In addition, the radial-SVM sensitivity analysis was limited to BNCI2014-004 and did not constitute a nested hyperparameter search or test the same settings on Stieger2021. Similarly, the MMD-bandwidth sensitivity analysis showed dataset-dependent variations in retained-source sets and fixed-configuration accuracy; the fixed σ = 1.0 setting should therefore be treated as an implementation heuristic rather than a universally transferable scale. Fifth, the leave-one-session-out design compares each held-out target session with all remaining available sessions rather than a strictly chronological, past-only session archive, so the results should be interpreted as cross-validation evidence rather than a prospective chronological deployment analysis. Finally, although the primary Stieger2021 benchmark includes 62 matched subjects, this cohort remains modest compared to the variability likely to arise in larger longitudinal archives.

### 4.6 Future directions

Future work should evaluate these pipelines in strictly chronological and online settings, where the source archive grows incrementally as sessions arrive. Configuration selection could also incorporate information from unlabeled target predictions rather than relying only on a source-defined proxy task. Relevant quantities include prediction confidence and agreement across source-trained models, both of which address model selection without target labels (You et al., 2019). In addition, target pseudo-labels could be assigned when models agree (Saito et al., 2017), with a confidence threshold applied to exclude unstable assignments (Sohn et al., 2020).

Recent EEG representation-learning models address a distinct component of the learning problem. GRAM learns representations from raw EEG for classification and signal restoration (Li et al., 2025). Diffusion-based EEGDM frameworks instead learn EEG representations through generative denoising. Specifically, Puah et al. employed structured state-space diffusion pretraining for clinical EEG classification, whereas Wang et al. evaluated latent-diffusion representations across several downstream tasks, including motor-imagery classification (Puah et al., 2025; Wang et al., 2025). In comparison, this study instead holds the representation grid fixed and examines within-subject source retention and configuration selection. These approaches are therefore complementary rather than competing baselines. An advantageous direction for future work would be to apply CI-gated source selection directly to pretrained EEG representations. Other extensions include cross-subject transfer, explicit measures of session drift, and validation on larger longitudinal archives.

## Data Availability

Publicly available datasets were analyzed in this study. BNCI2014_004 is accessible through the MOABB library (Jayaram and Barachant, 2018), and Stieger2021 is available as described by (Stieger et al. 2021). The software associated with this study is publicly available on GitHub, including the MSDA-Bench benchmark dashboard (https://github.com/Yiming-S/MSDA-Bench), the CrossPython cross-session pipeline framework (https://github.com/Yiming-S/CrossPython), the DA4BCI R package (https://github.com/Yiming-S/DA4BCI), and the DA4BCI-Python package, distributed as da4bci (https://github.com/Yiming-S/DA4BCI-Python).
