# Supplementary material for: A confidence-gated source selection strategy for cross-session transfer in brain–computer interfaces
Source: Front Hum Neurosci. 2026 Aug 28;20:1895016. doi: 10.3389/fnhum.2026.1895016 (PMC13562021; doi:10.3389/fnhum.2026.1895016)
Supplement: Supplementary file 1 [file Data_Sheet_1.pdf]

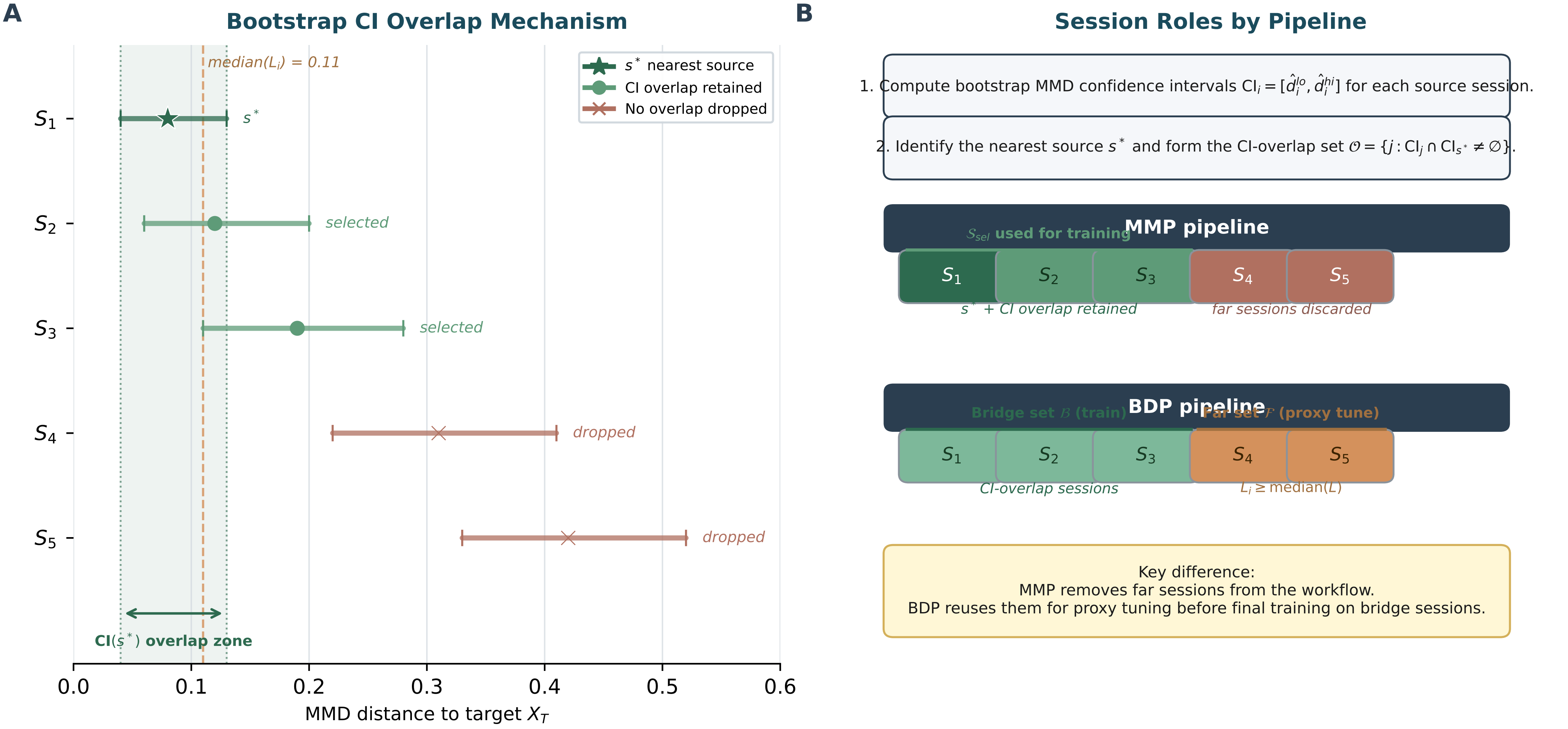
A
Bootstrap CI Overlap Mechanism
median(L_i) = 0.11
s^* nearest source
CI overlap retained
No overlap dropped
S_1
S_2
S_3
S_4
S_5
s^*
selected
selected
dropped
dropped
CI(s^*) overlap zone
0.0
0.1
0.2
0.3
0.4
0.5
0.6
MMD distance to target X_T
B
Session Roles by Pipeline
1. Compute bootstrap MMD confidence intervals $\mathrm{CI}_i = [\hat{d}_i^{lo}, \hat{d}_i^{hi}]$ for each source session.
2. Identify the nearest source s^* and form the CI-overlap set $\mathcal{O} = \{j : \mathrm{CI}_j \cap \mathrm{CI}_{s^*} \neq \emptyset\}$.
MMP pipeline
$\mathcal{S}_{sel}$ used for training
S_1
S_2
S_3
S_4
S_5
s^* + CI overlap retained
far sessions discarded
BDP pipeline
Bridge set $\mathcal{B}$ (train)
Far set $\mathcal{F}$ (proxy tune)
S_1
S_2
S_3
S_4
S_5
CI-overlap sessions
$L_i \geq$ median(L)
Key difference:
MMP removes far sessions from the workflow.
BDP reuses them for proxy tuning before final training on bridge sessions.
