# Supplementary material for: A confidence-gated source selection strategy for cross-session transfer in brain–computer interfaces
Source: Front Hum Neurosci. 2026 Aug 28;20:1895016. doi: 10.3389/fnhum.2026.1895016 (PMC13562021; doi:10.3389/fnhum.2026.1895016)
Supplement: Supplementary file 2 [file Data_Sheet_2.pdf]

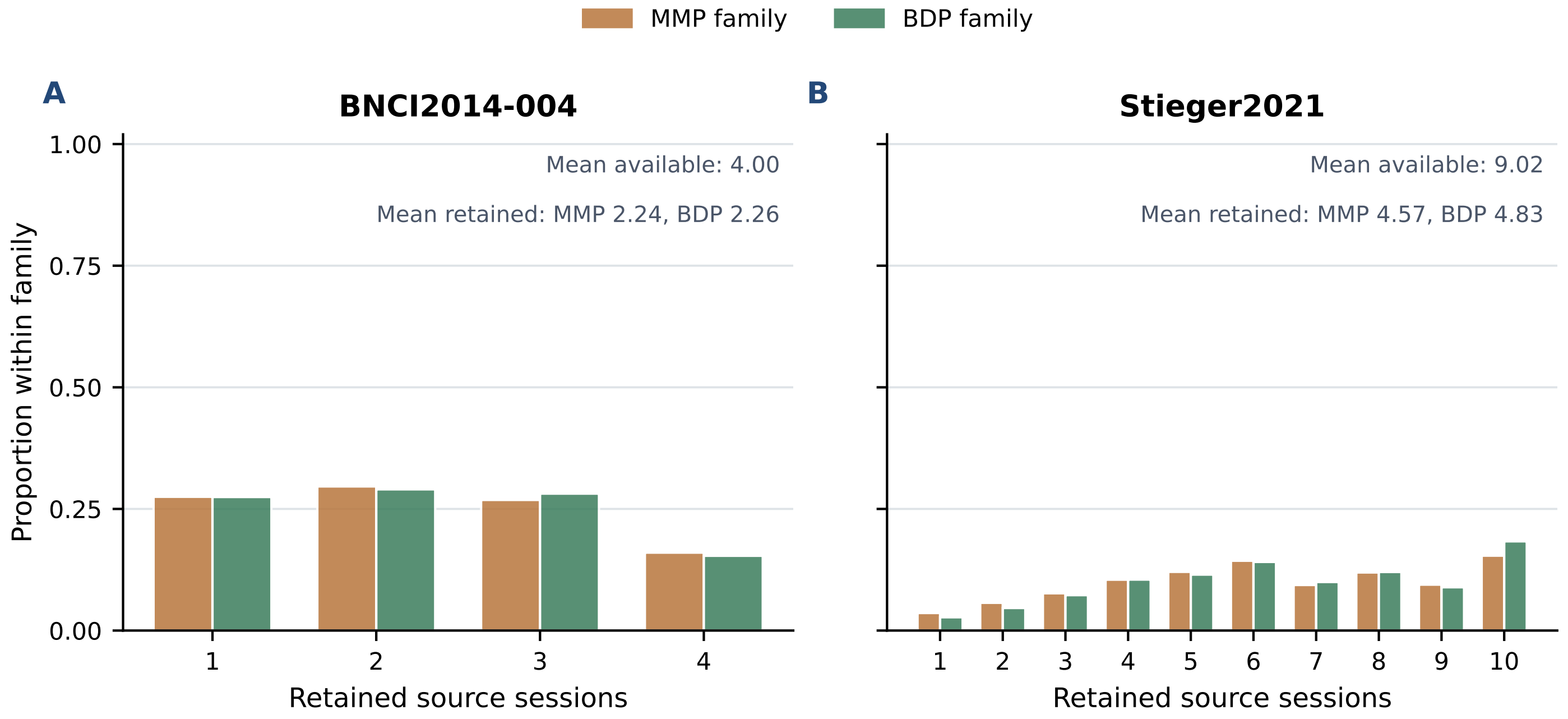
MMP family
BDP family
A
BNCI2014-004
B
Stieger2021
Proportion within family
1.00
0.75
0.50
0.25
0.00
Mean available: 4.00
Mean retained: MMP 2.24, BDP 2.26
Mean available: 9.02
Mean retained: MMP 4.57, BDP 4.83
1
2
3
4
5
6
7
8
9
10
Retained source sessions
Retained source sessions
