# Supplementary material for: A confidence-gated source selection strategy for cross-session transfer in brain–computer interfaces
Source: Front Hum Neurosci. 2026 Aug 28;20:1895016. doi: 10.3389/fnhum.2026.1895016 (PMC13562021; doi:10.3389/fnhum.2026.1895016)
Supplement: Supplementary file 3 [file Data_Sheet_3.pdf]

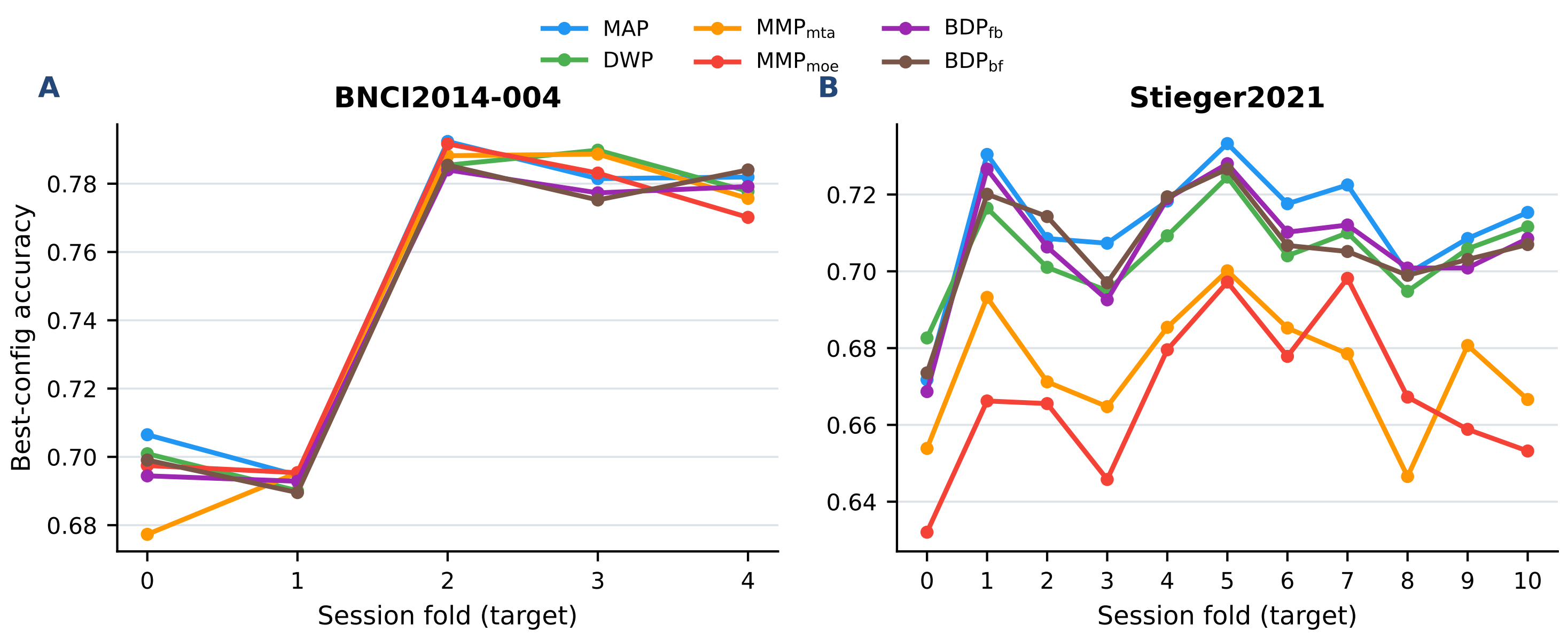

MAP
DWP
MMPmta
MMPmoe
BDPfb
BDPbf
A
BNCI2014-004
B
Stieger2021
Best-config accuracy
0.68
0.70
0.72
0.74
0.76
0.78
0.64
0.66
0.68
0.70
0.72
0
1
2
3
4
5
6
7
8
9
10
Session fold (target)
Session fold (target)
